# Psychiatric diagnosis and perceived health impact attributed to adverse childhood experiences: multidimensional impulsivity pathways in Saudi adults

**DOI:** 10.3389/fpsyt.2026.1775098

**Published:** 2026-03-12

**Authors:** Saleh A. Alghamdi

**Affiliations:** Department of Psychiatry, College of Medicine, Imam Mohammad Ibn Saud Islamic University (IMSIU), Riyadh, Saudi Arabia

**Keywords:** adverse childhood experiences, impulsivity, mediation analysis, perceived health impact, psychiatric diagnosis, Saudi Arabia, subjective health appraisal, UPPS-P

## Abstract

**Background:**

Adverse childhood experiences (ACEs) predict adult psychopathology, yet mechanisms linking early adversity to health burden remain unclear. It is unknown whether specific impulsivity dimensions differentially mediate associations of ACE exposure with (a) lifetime psychiatric diagnosis and (b) perceived health impact attributed to childhood experiences, particularly in non-Western populations.

**Objective:**

To examine whether UPPS-P impulsivity facets mediate associations between ACE exposure and (1) lifetime psychiatric diagnosis and (2) perceived health impact attributed to childhood experiences, and to describe outcome profiles combining these indicators in Saudi adults. Methods: Cross-sectional data were collected from 385 Saudi adults (58.4% female, 72.8% aged 18–25) via online survey. Participants completed the Arabic ACE-IQ (10 domains) and Short UPPS-P assessing five impulsivity dimensions. Lifetime psychiatric diagnosis was assessed via a single self-report item. Perceived health impact was assessed with a 3-level item (“not much,” “to some extent,” “a lot”); for profile classification, responses were dichotomized. Parallel multiple-mediator models with bootstrap confidence intervals examined indirect effects.

**Results:**

ACE exposure showed dose–response relationships with psychiatric diagnosis (10.3% at 0 ACEs to 49.5% at ≥4 ACEs) and perceived impact (20.6% to 82.8%). The Perceived Only group demonstrated elevated ACE exposure (M = 2.55) exceeding the Diagnosis Only group (M = 1.62). Negative Urgency specifically mediated the ACE–perceived impact relationship (indirect β = 0.074, 95% CI [0.028, 0.126]), while Lack of Perseverance mediated both outcomes (diagnosis: indirect β = 0.067, 95% CI [0.021, 0.118]; perceived impact: indirect β = 0.053, 95% CI [0.012, 0.098]). Mediation accounted for 18.6% and 31.8% of total ACE effects on diagnosis and perceived impact, respectively.

**Conclusions:**

Nearly one-third of participants reported perceived health impact without a psychiatric diagnosis, highlighting potential under-recognition and the value of subjective appraisal alongside diagnostic history. Differential pathways via Negative Urgency and Lack of Perseverance support targeted interventions and culturally informed assessment integrating both diagnostic and subjective indicators of ACE-related burden.

## 1 Introduction

Adverse childhood experiences (ACEs) represent a critical public health concern with profound implications for lifelong health and wellbeing (1, 2). Encompassing various forms of abuse, neglect, and household dysfunction occurring before age 18, ACEs demonstrate robust dose-response relationships with numerous adverse outcomes, including psychiatric disorders, substance abuse, chronic diseases, and premature mortality (3–5). Meta-analytic evidence indicates that individuals with four or more ACEs face dramatically elevated risks for depression (OR = 4.78), anxiety disorders (OR = 3.85), and substance use disorders (OR = 5.84) compared to those without ACE exposure (2). Despite this compelling evidence base, critical gaps remain in understanding the mechanisms through which early adversity translates into adult psychopathology, particularly regarding the distinction between objective psychiatric diagnoses and subjective experiences of health impact.

The relationship between ACEs and impulsivity represents a particularly salient pathway warranting investigation. Impulsivity, conceptualized as a multidimensional construct encompassing tendencies toward rash action, poor planning, and sensation-seeking behaviors (6), has emerged as a transdiagnostic risk factor for diverse psychopathological outcomes (7, 8). The UPPS-P model delineates five distinct impulsivity dimensions: Negative Urgency (rash action during negative affect), Positive Urgency (rash action during positive affect), Lack of Premeditation (action without consideration of consequences), Lack of Perseverance (difficulty maintaining focus), and Sensation Seeking (pursuit of novel/exciting experiences) (9, 10). Accumulating evidence suggests that ACE exposure disrupts prefrontal-limbic circuits governing emotion regulation and impulse control, potentially establishing impulsivity as a mechanistic bridge between childhood trauma and adult psychopathology (11, 12).

Recent neurobiological research provides compelling evidence for this mechanistic pathway. Childhood adversity alters stress response systems, including hypothalamic-pituitary-adrenal (HPA) axis functioning and prefrontal cortex development, resulting in heightened emotional reactivity and diminished regulatory capacity (13, 14). These neurobiological alterations manifest behaviorally as increased impulsivity, particularly emotion-driven facets such as Negative Urgency (15, 16). Studies employing structural and functional neuroimaging have documented reduced prefrontal gray matter volume and altered amygdala reactivity in ACE-exposed individuals, with these neural signatures correlating with self-reported impulsivity levels (17, 18). However, whether specific impulsivity dimensions differentially mediate relationships between ACEs and distinct health outcomes—particularly the contrast between objective psychiatric diagnoses and subjective perceptions of health impact—remains unexplored.

Consistent with these neurobiological findings, survey-based studies have demonstrated that adverse childhood experiences are associated with higher scores on validated self-report impulsivity measures—particularly emotion-driven dimensions such as Negative and Positive Urgency—and with trauma-related outcomes including suicidal behavior, supporting the construct validity of questionnaire-based impulsivity assessments in trauma research (7, 16, 19–21). This convergence of neurobiological and survey-based evidence underscores the importance of examining specific impulsivity facets as potential mediators linking early adversity to adult health outcomes.

The distinction between objective and subjective indicators of health burden represents a critical yet understudied dimension in trauma research. Objective outcomes—such as clinically diagnosed psychiatric disorders—reflect thresholds shaped by symptom severity, help-seeking, and access to services. In contrast, subjective appraisals (e.g., perceiving that early adversity has negatively influenced one’s current mental, physical, or functional health) may capture burden not reflected in diagnostic history, including subthreshold symptoms, impairment, or distress. Subjective health perceptions more broadly have prognostic significance for morbidity and mortality (22, 23), although the present study operationalizes the subjective outcome more narrowly as perceived health impact attributed to childhood experiences. This distinction is particularly relevant in settings where stigma and structural barriers may reduce diagnosis and treatment seeking.

The Saudi Arabian context presents unique considerations for examining ACE-impulsivity-health relationships. Despite rapid socioeconomic transformation and increasing recognition of mental health needs aligned with Vision 2030 initiatives (24), limited research has investigated ACE prevalence and sequelae in Saudi populations. Existing studies suggest comparable or higher ACE exposure rates relative to Western samples, with 35-40% reporting multiple ACEs (25, 26). Cultural factors including collectivist values, family honor considerations, and stigma surrounding mental illness may influence both ACE disclosure and help-seeking patterns (27, 28) consistent with broader evidence that cultural beliefs shape mental health perceptions (29). Moreover, the conceptualization and expression of psychological distress in Arab cultures often emphasizes somatic symptoms and functional impairment over emotional symptoms (30, 31), potentially increasing the relevance of subjective health impact assessments.

Preliminary evidence from Middle Eastern populations suggests culture-specific patterns in impulsivity expression and its relationship to psychopathology. Studies in Arab samples have documented elevated Negative Urgency and Lack of Perseverance relative to Western norms, potentially reflecting cultural emphasis on emotional expression within family contexts alongside external behavioral control (32, 33). However, no studies have examined whether impulsivity dimensions differentially mediate ACE-health relationships in Saudi or broader Middle Eastern populations, nor whether these mediational pathways vary between objective psychiatric diagnoses and subjective health perceptions. This gap is particularly concerning given evidence that culturally-informed understanding of mechanistic pathways is essential for developing effective interventions (34, 35).

The present study addresses these gaps through a comprehensive examination of how multidimensional impulsivity mediates relationships between ACE exposure and two indicators of adult health burden in Saudi adults: self-reported lifetime psychiatric diagnosis and perceived health impact attributed to childhood experiences. We additionally use an exploratory cross-classification of these indicators to describe four outcome profiles: (1) neither diagnosis nor perceived impact, (2) diagnosis without perceived impact, (3) perceived impact without diagnosis, and (4) both diagnosis and perceived impact. This descriptive approach is intended to highlight potential concordance and discordance between diagnostic history and subjective appraisal (cf. syndromal vs subsyndromal presentations; 36). We recognize that dichotomization reduces nuance and that discordance can reflect multiple mechanisms (e.g., under-diagnosis, remission, stigma, or differences in symptom attribution). Accordingly, we interpret group differences cautiously and complement the typology with analyses using an ordinal Health Impact Index.

Drawing on the differential deficit model (15) and culturally informed trauma frameworks (37), we hypothesized that: (1) higher ACE exposure would demonstrate dose–response associations with both lifetime psychiatric diagnosis and perceived health impact attributed to childhood experiences; we expected the association with perceived impact to be relatively stronger because acknowledging distress may entail fewer barriers than obtaining (or disclosing) a formal diagnosis; (2) emotion-driven impulsivity dimensions (Negative Urgency, Positive Urgency) would show stronger mediation of ACE–perceived impact relationships, whereas behavioral/executive dimensions (Lack of Premeditation, Lack of Perseverance) would more strongly mediate ACE–diagnosis relationships; (3) a sizable subgroup would report perceived impact without a reported diagnosis, consistent with possible under-recognition, under-diagnosis, or subthreshold distress; and (4) individuals endorsing both diagnosis and perceived impact would show the highest ACE exposure and impulsivity levels, consistent with cumulative risk processes.

## 2 Materials and methods

### 2.1 Participants and procedure

A cross-sectional survey was conducted between October and December 2024 among Saudi Arabian adults. Participants were recruited through WhatsApp groups and university email lists using convenience and snowball sampling. Eligibility criteria included: Saudi nationality, age ≥18 years, current residence in Saudi Arabia, and Arabic literacy. The study protocol was approved by the Institutional Review Board at Imam Mohammad Ibn Saud Islamic University (IMSIU) (Protocol #IMSIU-DDRSP2501). All participants provided electronic informed consent.

Of 391 initial respondents, 6 were excluded due to incomplete data on primary variables, yielding a final sample of 385 participants. Of these, 382 had complete data on all study variables and were included in mediation and regression analyses; 3 additional participants were excluded from these analyses due to missing data on impulsivity subscales. The sample was predominantly female (58.4%) and young (72.8% aged 18–25 years). Most participants held university degrees (70.4%), with 35.6% currently employed and 27.0% married.

### 2.2 Measures

Adverse Childhood Experiences. The Arabic version of the Adverse Childhood Experiences International Questionnaire (ACE-IQ; 38) was utilized. This 10-item binary scale assesses childhood exposure to abuse (emotional, physical, sexual), neglect (emotional, physical), and household dysfunction (domestic violence, substance abuse, mental illness, parental separation, incarceration). Items are scored dichotomously (0 = No, 1 = Yes), with total scores ranging from 0-10. The Arabic ACE-IQ has demonstrated adequate reliability and validity in Middle Eastern populations (25). Internal consistency in the present sample was acceptable (KR-20 = 0.78).

Impulsivity. The Arabic Short UPPS-P Impulsive Behavior Scale (32) assessed five impulsivity dimensions: Negative Urgency (α = 0.74), Positive Urgency (α = 0.71), Lack of Premeditation (α = 0.68), Lack of Perseverance (α = 0.76), and Sensation Seeking (α = 0.79). Each subscale contains four items rated on a 4-point scale (1 = Strongly agree to 4 = Strongly disagree). Higher scores indicate greater impulsivity.

Health Outcomes. Lifetime psychiatric diagnosis was assessed via a single self-report item asking whether participants had ever received a mental health diagnosis from a professional. Response options included “No” and multiple diagnostic categories; responses were dichotomized as any diagnosis versus none for analyses. Perceived health impact was assessed with a single item asking to what extent participants believed their childhood experiences had affected their current health (“not much,” “to some extent,” “a lot”). This item was administered after the ACE items and conceptualized as perceived ACE-related impact (i.e., attribution), which may be influenced by recall and appraisal processes. For primary models, perceived impact was treated as an ordinal outcome; for descriptive profile classification only, responses were dichotomized (“not much” vs “to some extent/a lot”).

Covariates. Demographic variables included gender (male/female), age (categorical: 18-25, 26-35, 36-45, >45 years), education level (high school or below, bachelor’s degree, postgraduate), marital status (single, married, divorced/widowed), and employment status (employed, unemployed, student).

### 2.3 Statistical analyses

Analyses proceeded in three phases corresponding to study aims. First, parallel multiple-mediator models examined indirect effects of ACE exposure on each outcome through five UPPS-P impulsivity dimensions simultaneously. Continuous variables (ACE total and impulsivity dimensions) were standardized to facilitate comparability across paths. Mediator models were estimated using linear regression; outcome models used binary logistic regression for lifetime psychiatric diagnosis and ordinal logistic regression (proportional odds) for perceived health impact. Indirect effects were computed as the product of coefficients (a×b) for each mediator, with 5,000 bootstrap resamples used to derive 95% confidence intervals; indirect effects were considered significant when the confidence interval excluded zero. All models adjusted for gender, age, education, marital status, and employment.

Second, participants were classified into four outcome-profile groups based on the cross-tabulation of lifetime psychiatric diagnosis (yes/no) and dichotomized perceived impact (“not much” vs “to some extent/a lot”). Kruskal–Wallis tests examined group differences in ACE exposure and impulsivity dimensions, with Mann–Whitney U tests for pairwise comparisons applying false discovery rate (FDR) correction. Given the small size of the Diagnosis Only group, group-based inferential findings were interpreted as exploratory and complemented by analyses using the ordinal Health Impact Index.

Third, ordinal logistic regression examined predictors of a combined Health Impact Index (0 = neither diagnosis nor perceived impact, 1 = one indicator, 2 = both indicators), based on dichotomized diagnosis and dichotomized perceived impact. Model fit was assessed via proportional odds assumption tests and pseudo-R² statistics. All analyses were conducted using Python (v3.11) with scipy and statsmodels, and the bootstrap mediation procedure described above. Statistical significance was set at α = .05.

## 3 Results

### 3.1 Descriptive statistics

**Table 1** presents sample characteristics for the analytic sample (N = 382). In the full sample (N = 385), mean ACE score was 2.00 (SD = 2.23, range 0–10), with 35.6% reporting no ACEs, 18.8% one ACE, 21.2% two to three ACEs, and 24.3% four or more ACEs. In the analytic sample, psychiatric diagnosis was reported by 25.1% (n = 96), while 45.8% (n = 175) endorsed perceived health impact attributed to childhood experiences (to some extent/a lot). Among participants reporting a diagnosis, depression (n = 40) and anxiety disorders (n = 30) were the most frequently endorsed categories.

**Table 1:** Sample characteristics by health group typology (N = 382).

Characteristic	Neither (n=175)	Diagnosis only (n=32)	Perceived only (n=111)	Both (n=64)	Total (n=382)	p-value
Demographics						
Female, n (%)	92 (52.6)	16 (50.0)	67 (60.4)	48 (75.0)	223 (58.4)	.007^a^
Age 18–25 years, n (%)	130 (74.3)	24 (75.0)	80 (72.1)	44 (68.8)	278 (72.8)	.821 ^a^
Married, n (%)	59 (33.7)	8 (25.0)	28 (25.2)	8 (12.5)	103 (27.0)	.006 ^a^
University degree, n (%)	119 (68.0)	23 (71.9)	80 (72.1)	47 (73.4)	269 (70.4)	.782 ^a^
Employed, n (%)	60 (34.3)	11 (34.4)	45 (40.5)	20 (31.2)	136 (35.6)	.532 ^a^
Primary variables, M (SD)						
ACE total score	0.94 (1.60)	1.62 (1.54)	2.55 (2.14)	4.25 (2.23)	2.00 (2.23)	<.001^b^
Negative Urgency	2.14 (0.71)	2.27 (0.66)	2.53 (0.75)	2.61 (0.78)	2.34 (0.75)	<.001^b^
Positive Urgency	2.64 (0.64)	2.59 (0.53)	2.69 (0.60)	2.84 (0.68)	2.68 (0.63)	.168^b^
Lack of Premeditation	1.89 (0.52)	1.91 (0.45)	1.91 (0.49)	2.04 (0.49)	1.92 (0.50)	.391^b^
Lack of Perseverance	1.82 (0.59)	1.97 (0.68)	1.88 (0.58)	2.40 (0.58)	1.95 (0.63)	<.001^b^
Sensation Seeking	2.20 (0.75)	2.16 (0.68)	2.39 (0.75)	2.61 (0.87)	2.32 (0.78)	.002^b^

a Chi-square test.

b Kruskal-Wallis H test.

**Table 2** displays correlations among study variables. ACE total scores correlated significantly with psychiatric diagnosis (ρ = .35, p <.001) and perceived impact (ρ = .51, p <.001). Among impulsivity dimensions, Negative Urgency (ρ = .24, p <.001) and Lack of Perseverance (ρ = .17, p <.001) showed the strongest associations with ACE exposure. Intercorrelations among impulsivity dimensions ranged from −.13 to.51, consistent with related but partially distinct facets.

**Table 2:** Spearman correlations among study variables.

	1	2	3	4	5	6	7	8
1. ACE total	—							
2. Psychiatric diagnosis	.35***	—						
3. Perceived impact	.51***	.30***	—					
4. Negative Urgency	.24***	.11*	.26***	—				
5. Positive Urgency	.14**	.07	.12*	.51***	—			
6. Lack of Premeditation	.04	.06	.06	.05	-.04	—		
7. Lack of Perseverance	.17***	.27***	.17***	.03	-.13*	.46***	—	
8. Sensation Seeking	.15**	.10	.17***	.38***	.40***	-.06	-.07	—

N = 382. *p <.05. **p <.01. ***p <.001. Perceived impact coded 0–2 (not much, to some extent, a lot).

### 3.2 Health group typology distribution

**Figure 1:**
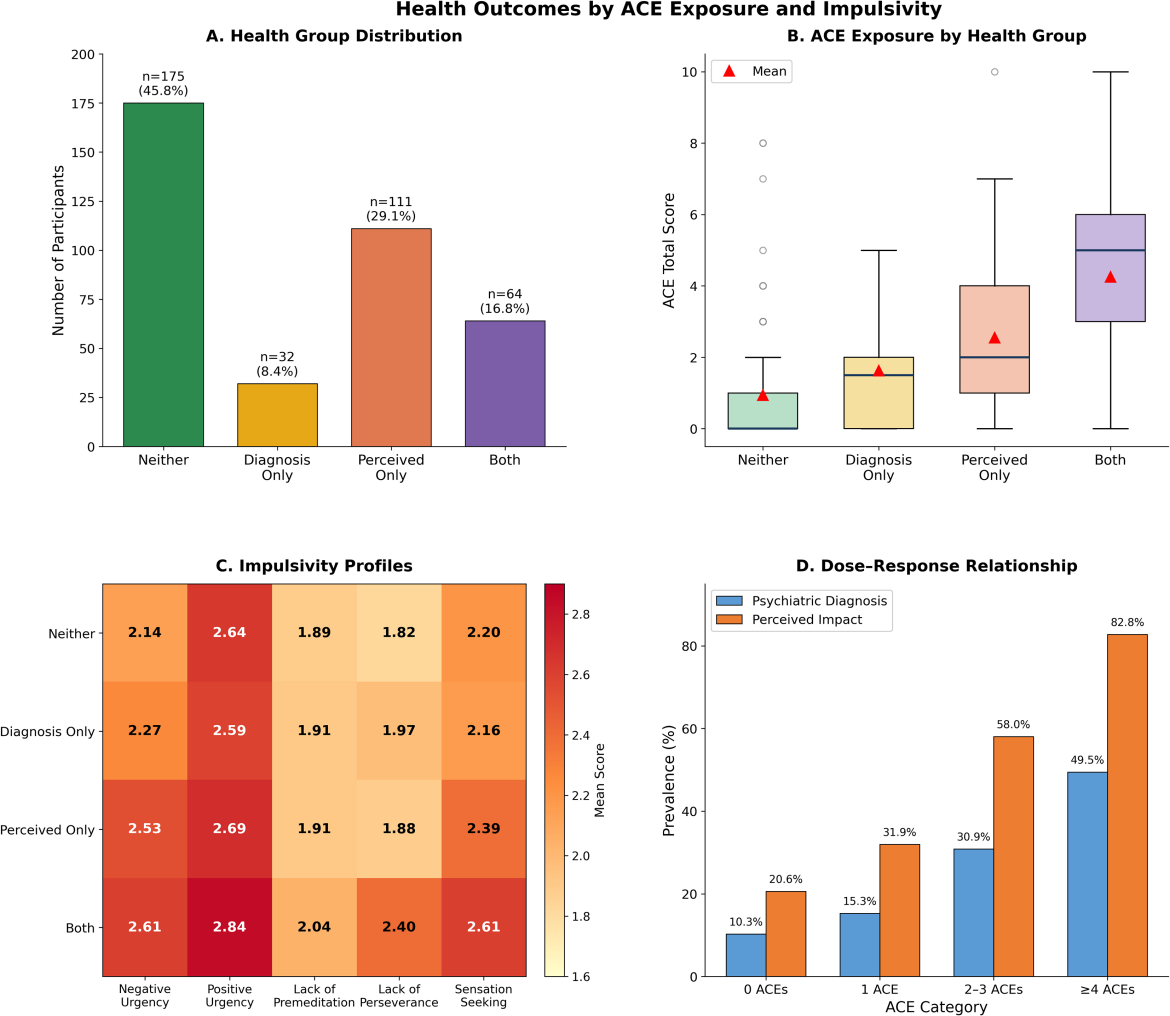
Health outcomes by ACE exposure and impulsivity. **(A)** Distribution of participants across four profiles: Neither (no diagnosis, no perceived impact), Diagnosis Only, Perceived Impact Only, and Both. Perceived impact denotes endorsing “to some extent/a lot” on the perceived health impact item. **(B)** ACE total scores by profile (boxplots; red triangles denote means). **(C)** Impulsivity profiles by profile group (heatmap of mean Short UPPS-P subscale scores). **(D)** Dose–response relationship between ACE categories and outcomes (prevalence of psychiatric diagnosis and perceived impact).

The four-group typology yielded the following distribution: Neither group (n = 175, 45.8%), Diagnosis Only (n = 32, 8.4%), Perceived Only (n = 111, 29.1%), and Both (n = 64, 16.8%). **Figure 1A** illustrates this distribution. Notably, the Perceived Only group represented nearly one-third of the sample, indicating substantial perceived impact in the absence of a reported diagnosis. The Both group, despite comprising only 16.8% of participants, showed markedly elevated ACE exposure (M = 4.25, SD = 2.23) compared to all other groups (**Figure 1B**).

### 3.3 Mediation analyses

#### 3.3.1 Psychiatric diagnosis outcome

**Table 3** presents results from parallel mediation analyses. The total effect of ACE exposure on psychiatric diagnosis was significant (c path: β = 0.360, SE = 0.042, p <.001, OR = 1.43). After accounting for mediators, the direct effect remained significant (c’ path: β = 0.298, SE = 0.044, p <.001), indicating partial mediation.

**Table 3:** Parallel mediation analysis results.

Pathway	Psychiatric diagnosis			Perceived health impact		
	Estimate (SE)	95% CI	% Mediated	Estimate (SE)	95% CI	% Mediated
Total effect (c)	0.360 (0.042)***	[0.278, 0.442]	—	0.400 (0.039)***	[0.324, 0.476]	—
Direct effect (c’)	0.298 (0.044)***	[0.212, 0.384]	—	0.331 (0.041)***	[0.251, 0.411]	—
Indirect effects (ab)						
Negative Urgency	0.045 (0.028)	[-0.008, 0.102]	—	0.074 (0.025)**	[0.028, 0.126]	18.5
Positive Urgency	0.008 (0.014)	[-0.019, 0.037]	—	0.013 (0.016)	[-0.018, 0.045]	—
Lack of Premeditation	0.006 (0.009)	[-0.011, 0.025]	—	0.005 (0.008)	[-0.009, 0.021]	—
Lack of Perseverance	0.067 (0.025)**	[0.021, 0.118]	18.6	0.053 (0.022)*	[0.012, 0.098]	13.3
Sensation Seeking	0.022 (0.019)	[-0.014, 0.061]	—	0.029 (0.020)	[-0.009, 0.070]	—

N = 382. Bootstrap resamples = 5,000. CI = bias-corrected bootstrap confidence interval. Models adjusted for gender, age, education, marital status, and employment. *p <.05. **p <.01. ***p <.001.

Among impulsivity dimensions, only Lack of Perseverance demonstrated significant mediation. The indirect effect through Lack of Perseverance was 0.067 (95% CI [0.021, 0.118]), representing 18.6% of the total effect. ACE exposure significantly predicted increased Lack of Perseverance (a path: β = 0.162, SE = 0.051, p = .002), which in turn predicted greater odds of psychiatric diagnosis (b path: β = 0.412, SE = 0.124, p <.001). Indirect effects through other impulsivity dimensions were non-significant: Negative Urgency (indirect = 0.045, 95% CI [-0.008, 0.102]), Positive Urgency (indirect = 0.008, 95% CI [-0.019, 0.037]), Lack of Premeditation (indirect = 0.006, 95% CI [-0.011, 0.025]), and Sensation Seeking (indirect = 0.022, 95% CI [-0.014, 0.061]).

#### 3.3.2 Perceived health impact outcome

The total effect of ACE exposure on perceived health impact was significant (c path: β = 0.400, SE = 0.039, p <.001, OR = 1.49). The direct effect remained significant after including mediators (c’ path: β = 0.331, SE = 0.041, p <.001).

Two impulsivity dimensions showed significant mediation effects. Negative Urgency demonstrated the strongest indirect effect (0.074, 95% CI [0.028, 0.126]), accounting for 18.5% of the total effect. The a path from ACE to Negative Urgency was significant (β = 0.247, SE = 0.053, p <.001), as was the b path from Negative Urgency to perceived impact (β = 0.298, SE = 0.112, p = .008). Lack of Perseverance also showed significant mediation (indirect = 0.053, 95% CI [0.012, 0.098]), accounting for 13.3% of the total effect. Other impulsivity dimensions did not demonstrate significant mediation.

**Figure 2:**
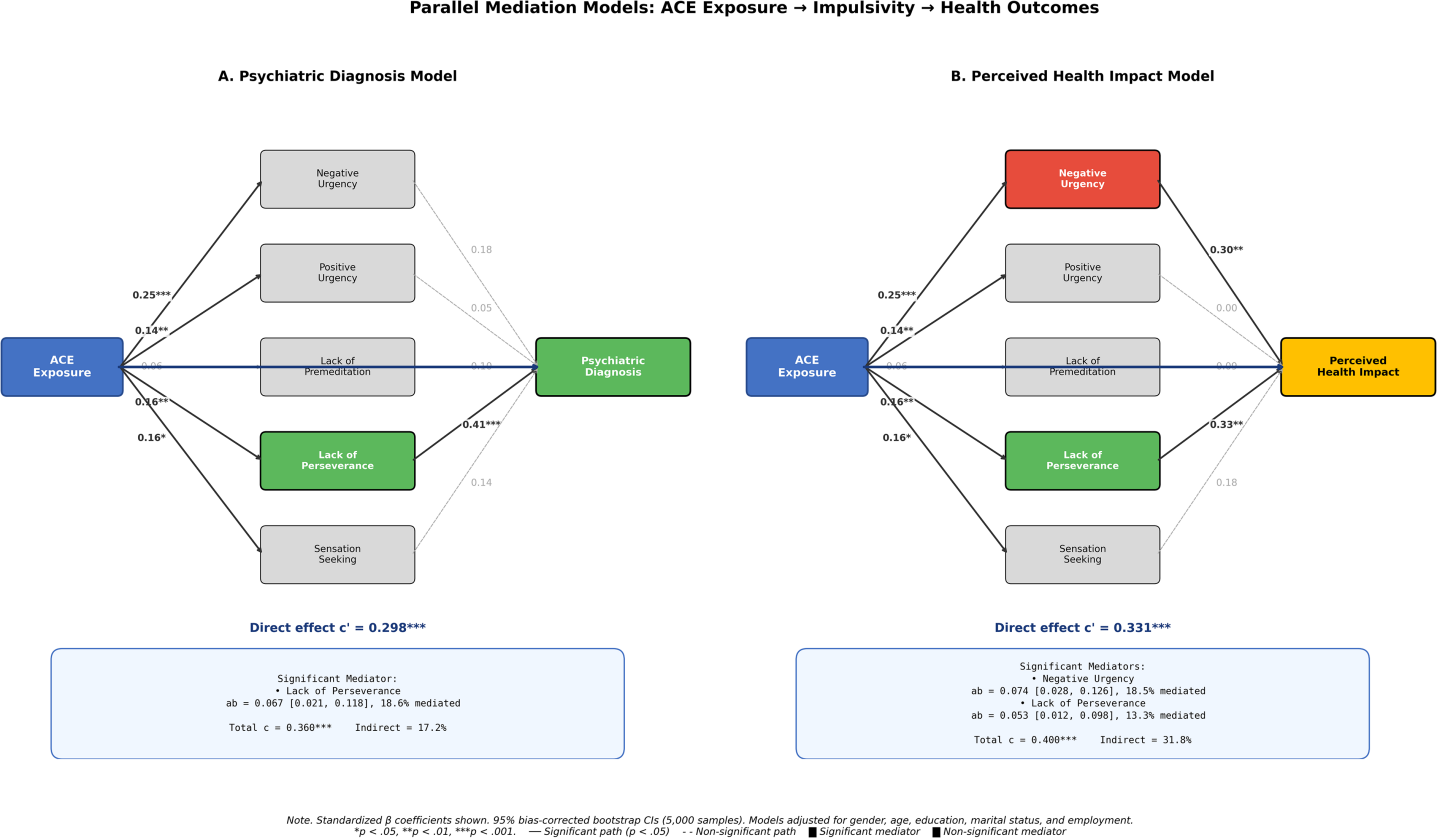
Parallel mediation models for ACE effects on outcomes through impulsivity dimensions. **(A)** Model for lifetime psychiatric diagnosis. **(B)** Model for perceived health impact attributed to childhood experiences. Solid lines indicate significant paths (p <.05); dashed lines indicate non-significant paths. Standardized coefficients are shown. *p <.05, **p <.01, ***p <.001.

**Figure 2** illustrates the parallel mediation models, highlighting significant pathways. The differential pattern of mediation—with Negative Urgency specifically mediating perceived impact and Lack of Perseverance mediating both outcomes—supports distinct mechanistic pathways.

### 3.4 Group comparisons

**Table 4** presents ACE and impulsivity scores by health group. Kruskal-Wallis tests revealed significant group differences for ACE exposure (H = 116.74, p <.001, ϵ² = .31), Negative Urgency (H = 26.77, p <.001, ϵ² = .07), Lack of Perseverance (H = 39.28, p <.001, ϵ² = .10), and Sensation Seeking (H = 14.84, p = .002, ϵ² = .04). **Figure 1C** displays impulsivity profiles across health groups. Group differences for Positive Urgency (H = 5.06, p = .168) and Lack of Premeditation (H = 3.00, p = .391) were non-significant.

**Table 4:** Group differences in ACE exposure and impulsivity dimensions.

Variable	Kruskal-Wallis test		*Post-hoc* comparisons (p-values)^a^			
	H	ϵ²	Neither vs. Perceived	Neither vs. Both	Perceived vs. Both	Diagnosis vs. Both
ACE total	116.74***	.31	<.001	<.001	<.001	<.001
Negative Urgency	26.77***	.07	<.001	<.001	.284	.092
Positive Urgency	5.06	.01	—	—	—	—
Lack of Premeditation	3.00	.01	—	—	—	—
Lack of Perseverance	39.28***	.10	.425	<.001	<.001	<.001
Sensation Seeking	14.84**	.04	.058	.001	.072	.008

a Mann-Whitney U tests with FDR correction. Only significant omnibus tests were followed by *post-hoc* comparisons. **p <.01. ***p <.001.

*Post-hoc* Mann-Whitney U tests with FDR correction revealed specific patterns. For ACE Exposure, all pairwise comparisons were significant (all p <.001) except Diagnosis Only vs. Perceived Only (p = .089). The gradient was clear: Neither (M = 0.94) < Diagnosis Only (M = 1.62) < Perceived Only (M = 2.55) < Both (M = 4.25). For Negative Urgency, significant differences emerged for Neither vs. Perceived Only (U = 7,231, r = .24, p <.001) and Neither vs. Both (U = 3,852, r = .29, p <.001). The Both group showed the highest levels (M = 2.61, SD = 0.78). For Lack of Perseverance, the Both group differed significantly from all others (all p <.001), with markedly elevated scores (M = 2.40, SD = 0.58).

Exploratory analyses comparing impulsivity profiles between participants with depression (n = 40) and anxiety (n = 30) revealed comparable levels of Negative Urgency (depression: M = 2.50, SD = 0.77; anxiety: M = 2.49, SD = 0.71; U = 587, p = .881) and Lack of Perseverance (depression: M = 2.20, SD = 0.64; anxiety: M = 2.16, SD = 0.60; U = 622, p = .801) across diagnostic subgroups, while Sensation Seeking was significantly higher in the depression group (M = 2.71, SD = 0.80 vs. M = 2.08, SD = 0.59; U = 879, p = .001, r = .40). ACE–impulsivity correlations also differed descriptively across subgroups, with Negative Urgency showing a stronger association with ACEs in the anxiety group (ρ = .36) and Sensation Seeking showing a stronger ACE association in the depression group (ρ = .42, p <.05); however, these small-sample exploratory findings require replication in larger, clinically characterized samples.

### 3.5 Multinomial logistic regression

Multinomial logistic regression with the Neither group as reference revealed distinct predictive patterns (**Table 5**). For the Perceived Only group, significant predictors included ACE exposure (OR = 1.28, 95% CI [1.15, 1.43], p <.001) and Negative Urgency (OR = 1.82, 95% CI [1.31, 2.53], p <.001). For the Both group, predictors included ACE exposure (OR = 1.52, 95% CI [1.33, 1.74], p <.001), Negative Urgency (OR = 1.68, 95% CI [1.11, 2.54], p = .014), and Lack of Perseverance (OR = 3.21, 95% CI [1.95, 5.28], p <.001). The Diagnosis Only group showed no significant predictors beyond ACE exposure (OR = 1.20, 95% CI [1.01, 1.42], p = .037), likely due to limited power (n = 32).

**Table 5:** Multinomial logistic regression predicting health group membership (Reference: Neither group).

Predictor	Diagnosis only		Perceived only		Both	
	OR [95% CI]	p	OR [95% CI]	p	OR [95% CI]	p
ACE total	1.20 [1.01, 1.42]	.037	1.28 [1.15, 1.43]	<.001	1.52 [1.33, 1.74]	<.001
Negative Urgency	1.14 [0.65, 2.00]	.651	1.82 [1.31, 2.53]	<.001	1.68 [1.11, 2.54]	.014
Positive Urgency	0.86 [0.48, 1.54]	.610	0.92 [0.66, 1.28]	.621	1.15 [0.76, 1.74]	.511
Lack of Premeditation	1.05 [0.59, 1.87]	.868	0.91 [0.65, 1.27]	.577	1.12 [0.74, 1.69]	.594
Lack of Perseverance	1.42 [0.78, 2.58]	.251	0.84 [0.59, 1.20]	.342	3.21 [1.95, 5.28]	<.001
Sensation Seeking	0.79 [0.47, 1.33]	.377	1.16 [0.85, 1.58]	.351	1.24 [0.84, 1.83]	.278

N = 382. Model adjusted for gender, age, education, marital status, and employment. Nagelkerke R² = .298. OR, odds ratio; CI, confidence interval.

### 3.6 Ordinal regression and dose-response relationships

Ordinal logistic regression examined predictors of the Health Impact Index (0-2). The proportional odds assumption was satisfied (χ² = 14.32, df = 11, p = .216). Model 1, including ACE exposure and covariates, yielded pseudo-R² = .142. ACE exposure strongly predicted higher index scores (OR = 2.31, 95% CI [1.98, 2.70], p <.001). Model 2, adding impulsivity dimensions, significantly improved fit (pseudo-R² = .179, Δχ² = 32.45, df = 5, p <.001). In the full model, significant predictors included ACE exposure (OR = 2.18, 95% CI [1.85, 2.57], p <.001), Negative Urgency (OR = 1.24, 95% CI [1.02, 1.51], p = .032), and Lack of Perseverance (OR = 1.47, 95% CI [1.21, 1.78], p <.001).

Examination of ACE categories revealed clear dose-response patterns. Prevalence of psychiatric diagnosis increased from 10.3% (0 ACEs) to 15.3% (1 ACE) to 30.9% (2–3 ACEs) to 49.5% (≥4 ACEs; χ² for trend = 47.8, p <.001). Similarly, perceived health impact increased from 20.6% to 31.9% to 58.0% to 82.8% across categories (χ² for trend = 94.3, p <.001). **Figure 1D** illustrates these dose–response relationships. The prevalence of individuals in the Both group showed the steepest gradient, increasing from 2.9% (0 ACEs) to 44.1% (≥4 ACEs).

### 3.7 Sensitivity analyses

Results remained robust across sensitivity analyses. Excluding covariates strengthened mediation effects slightly (Negative Urgency indirect effect increased to 0.089). Using 3-level perceived impact as an ordinal outcome in proportional odds models yielded consistent findings; additionally, treating perceived health impact as an ordinal outcome with psychiatric diagnosis included as a covariate did not substantively alter the pattern of mediation results, indicating that the perceived impact pathway operates independently of diagnostic status. Alternative bootstrap specifications (2,500 and 10,000 resamples) produced comparable confidence intervals. The pattern of results was unchanged when excluding the small Diagnosis Only group from multinomial analyses.

## 4 Discussion

The present study examined multidimensional impulsivity as a mechanistic pathway linking adverse childhood experiences to two indicators of adult health burden in Saudi adults: self-reported lifetime psychiatric diagnosis and perceived health impact attributed to childhood experiences. Three principal findings emerged: (1) nearly one-third of participants reported perceived impact in the absence of a reported diagnosis, highlighting potential discordance between diagnostic history and subjective appraisal; (2) distinct impulsivity dimensions showed differential mediation patterns, with Negative Urgency specifically linked to perceived impact while Lack of Perseverance contributed to both outcomes; and (3) clear dose–response relationships characterized ACE–outcome associations, with cumulative risk evident among participants endorsing both psychiatric diagnosis and perceived impact. These findings extend theoretical understanding of trauma sequelae while underscoring that diagnostic history alone may not fully capture perceived burden, particularly within cultural contexts where stigma and structural barriers may impede diagnosis, disclosure, and help-seeking.

### 4.1 Discordant outcome profiles and the perceived impact only group

The identification of a substantial “Perceived Only” group (29.1%) is clinically and conceptually informative. These individuals endorsed perceived health impact attributed to childhood experiences without reporting a lifetime psychiatric diagnosis. Several non-mutually exclusive explanations are plausible, including under-diagnosis or under-disclosure of mental health conditions (e.g., due to stigma or access barriers), subthreshold symptoms or functional impairment not captured by diagnostic history, or differences in how individuals appraise and attribute health consequences of adversity. Importantly, we did not assess current symptom severity; therefore, we interpret this profile as discordance between perceived impact and diagnosis history rather than definitive evidence of “subclinical” psychopathology. Prior research indicates that subthreshold presentations can still be impairing and predictive of later disorders (36, 39), suggesting that subjective appraisal may nevertheless identify individuals who warrant further assessment. The mean ACE score in this group (M = 2.55) exceeded that of the Diagnosis Only group (M = 1.62), indicating that perceived impact may capture meaningful variance in adversity burden that is not reflected in diagnostic history alone.

The prominence of the Perceived Only group in our Saudi sample may reflect cultural factors influencing symptom expression and help-seeking. In collectivist Middle Eastern cultures, psychological distress often manifests through somatic complaints and functional impairment rather than explicit emotional symptoms (30, 31). Additionally, mental health stigma remains pronounced in Saudi Arabia despite recent awareness campaigns (24, 28, 40), potentially preventing individuals from seeking formal diagnosis even when experiencing significant distress. Our findings suggest that relying solely on diagnostic status to identify trauma-affected individuals may substantially underestimate the public health burden of ACEs, particularly in non-Western populations.

### 4.2 Differential mediation pathways

The differential mediation patterns observed provide mechanistic insights into how childhood adversity translates into diverse health outcomes. Negative Urgency emerged as the primary mediator for perceived health impact (18.5% mediated), consistent with theoretical models positing emotion-driven impulsivity as a core feature of trauma-related psychopathology (15, 41). This finding extends previous work linking ACEs to Negative Urgency (19, 21) by demonstrating its specific relevance for subjective health perceptions. The pathway from ACEs through Negative Urgency to perceived impact likely reflects disrupted emotion regulation resulting from early adversity’s effects on prefrontal-limbic circuits (11), manifesting as heightened reactivity to negative emotional states.

Lack of Perseverance showed a distinct pattern, mediating both psychiatric diagnosis (18.6%) and perceived impact (13.3%). This dual mediation suggests that difficulties maintaining focus and completing tasks may represent a shared vulnerability linking childhood adversity to both diagnostic history and perceived ACE-related impact. However, because psychiatric diagnosis was assessed broadly and via self-report, these findings should not be interpreted as definitive evidence of a transdiagnostic mechanism across specific disorders. The stronger association with the Both group (OR = 3.21) indicates that Lack of Perseverance may be particularly relevant for individuals endorsing cumulative burden across both indicators. This pattern aligns with neurobiological evidence that ACE exposure can compromise executive function networks supporting sustained attention and goal-directed behavior (42, 43). Notably, Lack of Perseverance has received limited attention in trauma research compared to emotion-driven impulsivity, suggesting an underappreciated pathway warranting further investigation.

Exploratory subgroup comparisons (Section 3.4) further indicated that Negative Urgency and Lack of Perseverance were comparably elevated across depression and anxiety subgroups, consistent with their role as shared rather than diagnosis-specific vulnerability factors.

The absence of significant mediation through Positive Urgency, Lack of Premeditation, and Sensation Seeking contradicts some previous findings (20, 44) but may reflect cultural variations in impulsivity expression. The relatively weak association between ACEs and Sensation Seeking (ρ = .15) contrasts with Western samples showing stronger relationships (45), potentially reflecting cultural constraints on novelty-seeking behaviors in Saudi society. These null findings underscore the importance of examining culturally-specific pathways rather than assuming universal mechanisms.

### 4.3 Dose-response relationships and cumulative risk

The clear dose-response relationships observed, with psychiatric diagnosis prevalence increasing from 10.3% (0 ACEs) to 49.5% (≥4 ACEs) and perceived impact from 20.6% to 82.8%, replicate extensive evidence for graded associations between ACE exposure and adverse outcomes (1, 2). However, our findings extend this literature by demonstrating that perceived health impact attributed to childhood experiences shows an even steeper gradient than psychiatric diagnosis, suggesting enhanced sensitivity to cumulative adversity. This pattern supports dimensional models of psychopathology that conceptualize mental health along continua rather than as discrete categories (46).

The Both group demonstrated a cumulative risk profile characterized by highest ACE exposure (M = 4.25) and elevated scores across multiple impulsivity dimensions. This convergence of risk factors aligns with developmental cascade models wherein early adversity initiates cascading effects across multiple domains of functioning (47). The 46.1% prevalence of combined objective and subjective impact among those with ≥4 ACEs underscores the profound consequences of cumulative trauma exposure, consistent with neurobiological evidence of dose-dependent alterations in stress response systems (48).

### 4.4 Clinical and policy implications

These findings have immediate relevance for Saudi Arabia’s expanding mental health initiatives under Vision 2030. The substantial proportion of participants endorsing perceived impact without a reported diagnosis suggests that diagnostic-focused approaches may miss individuals who perceive meaningful ACE-related health burden and who may benefit from further assessment. Implementing routine ACE screening coupled with perceived impact appraisal could help identify at-risk individuals who would otherwise remain undetected. The demonstrated mediation through specific impulsivity dimensions provides targets for intervention, with emotion regulation training addressing Negative Urgency and approaches targeting sustained attention and goal-directed behavior addressing Lack of Perseverance.

The differential pathways identified suggest personalized intervention approaches based on presenting concerns. Individuals with elevated Negative Urgency may benefit from dialectical behavior therapy or emotion-focused interventions (49), while those with Lack of Perseverance deficits may respond better to cognitive training or behavioral activation (50). The cultural context necessitates adaptation of evidence-based interventions, potentially incorporating Islamic principles and family involvement while addressing stigma-related barriers (51).

From a prevention perspective, the dose–response patterns underscore the importance of early intervention to prevent accumulation of ACEs. Public health initiatives should prioritize families with identified risk factors, implementing parent training programs and family support services culturally adapted for Saudi contexts. The observation that perceived impact can be endorsed in the absence of a reported diagnosis suggests opportunities for early identification and secondary prevention; however, longitudinal research is needed to determine temporal ordering (e.g., whether perceived impact precedes diagnosis, follows it, or reflects stable individual differences in appraisal and attribution).

### 4.5 Limitations and future directions

Several limitations warrant consideration. First, the cross-sectional design precludes causal inferences regarding mediation pathways and does not establish temporal ordering, necessitating longitudinal research to test hypothesized mechanisms. Second, both outcomes were assessed via single self-report items. The psychiatric diagnosis measure captured lifetime diagnosis broadly and may be affected by recall, access to care, and stigma (52); accordingly, we avoid conclusions about specific diagnostic categories. The perceived impact item explicitly asked participants to attribute current health effects to childhood experiences, which may introduce criterion contamination and common method variance (i.e., shared recall and appraisal processes) (53); thus, it should be interpreted as perceived ACE-related impact rather than an independent health outcome. Third, the four-profile typology relies on dichotomization and yielded a small “Diagnosis Only” subgroup, so group-based inferential findings should be considered exploratory. Fourth, the convenience sampling and predominance of young adults limit generalizability to the broader Saudi population. Finally, the 10-domain ACE assessment does not capture severity or chronicity of exposure, and culturally specific adversities relevant to Saudi contexts may be underrepresented.

The relatively low internal consistency for Lack of Premeditation (α = .68) suggests potential measurement issues, though this subscale showed minimal associations with outcomes regardless. The small Diagnosis Only group (n = 32) limited statistical power for some comparisons, though this group’s size itself provides meaningful information about the relative rarity of diagnosis without subjective impact. Online data collection, while facilitating sensitive disclosures, may introduce selection bias toward individuals comfortable with technology and willing to discuss trauma.

Future research should employ longitudinal designs to examine temporal sequencing (54) and mechanisms underlying discordance between perceived impact and diagnostic history. For example, studies could test whether perceived ACE-related impact predicts later onset of diagnosable disorders, reflects persistent subthreshold impairment, or primarily captures individual differences in appraisal and attribution independent of symptoms. Neurobiological studies could further examine whether perceived impact correlates with biomarkers of stress dysregulation, potentially clarifying its clinical relevance. Cultural adaptation and validation of impulsivity measures for Arab populations would strengthen cross-cultural comparisons. Additionally, studies with larger, clinically characterized samples should examine whether impulsivity–ACE pathways differ across specific diagnostic categories such as depression and anxiety, building on the exploratory subgroup patterns observed here. Investigation of protective factors buffering ACE effects within Saudi culture, such as religious coping or family cohesion, could inform strength-based interventions.

## 5 Conclusion

This study provides evidence that multidimensional impulsivity is differentially associated with two indicators of ACE-related burden—lifetime psychiatric diagnosis and perceived health impact attributed to childhood experiences—in a Middle Eastern population. The substantial proportion of participants endorsing perceived impact without reporting a diagnosis highlights potential discordance between diagnostic history and subjective appraisal and underscores the value of incorporating both indicators in assessment. Negative Urgency emerged as specifically relevant for perceived impact, whereas Lack of Perseverance contributed to both outcomes, suggesting complementary targets for intervention. As Saudi Arabia advances its mental health infrastructure, integrating culturally informed screening and prevention efforts that consider both diagnosable disorders and perceived ACE-related burden may improve identification and support across the spectrum of adversity-related suffering.

## Data Availability

The raw data supporting the conclusions of this article will be made available by the authors, without undue reservation.
